# Host sex-specific parasites in a functionally dioecious fig: a preference way of adaptation to their hosts

**DOI:** 10.1002/ece3.682

**Published:** 2013-07-30

**Authors:** Qi Wang, Zi-Feng Jiang, Ning-xin Wang, Li-ming Niu, Zi Li, Da-Wei Huang

**Affiliations:** 1 College of Plant Protection, Shandong Agricultural UniversityTai'an, Shandong, 271018, China; 2 Cotton Research Center, Shandong Academy of Agricultural SciencesJinan, 250100, China; 3 Institute for Genomics and Systems Biology, The University of ChicagoChicago, Illinois 60615; 4 Tropical crops genetic resources instituteDanzhou, Hainan 571737, China; 5 Institute of Zoology, Chinese Academy of SciencesBeijing 100101, China

**Keywords:** Coevolution, dioecy, DNA barcoding, host shift, nonpollinating fig wasp

## Abstract

Host–parasites interaction is a common phenomenon in nature. Diffusive coevolution might maintain stable cooperation in a fig–fig wasps system, in which the exploiter might diversify their genotype, phenotype, or behavior as a result of competition with pollinator, whereas the figs change flower syconia, fruits thickness, and syconia structure. In functionally dioecious *Ficus auriculata*, male figs and female figs contain two types of florets on separate plant, and share high similarities in outside morphology. *Apocryptophagus* (Sycophaginae, Chalcidoidea, Hymenoptera) is one of few groups of nonpollinating fig wasps that can reproduce within both male and female figs. On the basis of the morphology and DNA barcoding, evidence from partial sequences of mitochondrial cytochrome c oxidase I and nuclear internal transcribed spacer 2, we found that there are two nonsibling *Apocryptophagus* species living on male and female *F. auriculata* figs, respectively. We estimated that these two species diverged about 19.2 million years ago. Our study suggests that the host shift from *Ficus variegate* or *Ficus prostrata* fig species to male figs is a preference way for *Apocryptophagus* wasps to adapt to the separation of sexual function in diecious figs. Furthermore, to escape the disadvantage or sanction impact of the host, the exploiter *Apocryptophagus* wasps can preferably adapt to exploiting each sex of the figs, by changing their oviposition, niche shift, and habitat.

## Introduction

The coevolution between hosts and their parasites or between mutualistic partners is common phenomenon in nature (Thrall et al. 2007; Elias et al. 2008). Compared with their free-living relatives, parasites or coevolved mutualistic partners often show dramatic changes in phenotype to adapt to their hosts or the other mutualistic partners (Mcleish et al. 2010). The changes in host/one mutual partners are often accompanied by the according changes in parasites or the other partners. The pollinating wasps and nonpollinating fig wasps (NPFW) on the same host figs constitute a classic example of the mutualistic and parasitic association in plant-insect coevolution (Weiblen 2003). Diffusive coevolution might maintain stable cooperation in a fig–fig wasps system, in which NPFW might diversify their genotype, phenotype, or behavior as a result of competition with pollinator, whereas the figs change flower syconia, fruits thickness, and syconia structure (Wang et al. 2010). It has been an ideal system for addressing an array of evolutionary ecology questions including sex allocation, precise adaptation (Weiblen 2002; Cook and Rasplus 2003; Molbo et al. 2003; Herre et al. 2008).

There are about 750 known fig species in the world (Berg 1989). About half of them are functionally dioecious and the other half are monoecious. In monoecious figs, seeds, pollinators and other NPFW are all produced in one fig (Kerdelhue et al. 2000). In functionally dioecious *Ficus* species, male figs (also known as gall figs) have short styles and produces pollinators that disperse fig pollen. Female figs (also known as seed figs) have longer styles that are too long for pollinator's ovipositors to reach the ovules and only produce seeds (Ganeshaiah 1995). However, in most of monoecious *Ficus* species, pollinator could possess oviposition in flowers with long styles (Nefdt and Compton 1996), for example, in *Ficus racemosa*, the spatial constraints of female flowers cannot prevent pollinators from ovipositing more eggs, and showed sufficiently negative correlation between host and pollinator when the local resource is saturated (Wang et al. 2008), so that asymmetric interaction exist between cooperative players to maintain stable cooperation (Wang et al. 2011). Once fig pollinators developed the ability to discriminate male and female figs and pursued their own benefits by only entering into male figs, the fig–fig pollinator mutualism would theoretically go extinct. Thus, male figs and female figs share high similarities in appearance under the strong selection of sexual mimicry except that they have dimorphic styles inside their figs (Weiblen 1999).

The separation of sexual function in dioecious figs seems to have enormous advantages to the fig–fig pollinator's mutualism (Weiblen et al. 2001). It facilitates exclusion of many NPFW in female figs. For most NPFW, they can only occur in male figs as they are dependent on the development of pollinator's larvae as food resource or the presence of pollinator to go through the fig development barrier. Only very few groups of NPFW such as *Apocryptophagus* can live inside male and female figs, independent of the absence of pollinators or other NPFW (Bouček 1988). These *Apocryptophagus* wasps have been shown to only produce 10 times less offspring in female figs in the lack of male figs in few fig species that have been investigated (Peng et al. 2005). Molecular phylogeny reconstruction revealed multiple transitions from monoecy to dioecy in the evolution of *Ficus* (Weiblen 2000; Jousselin et al. 2003). Thus, each transition from monoecy to dioecy will make female figs free of parasitism of their original NPFW. As the arm race is well known as the main theme of host–parasite interaction, is there any according change in NPFW to adapt to the separation of sexual function in dioecious figs?

In this study, we collected hundreds of *Apocryptophagus* wasp specimens from male and female figs of *Ficus auriculata*. By using the combination of morphological examination and DNA barcoding analyses, we found that there are two nonsibling *Apocryptophagus* species living on male and female *F. auriculata* figs, respectively. Our study suggests that host shift from other fig species (i.e., *Ficus variegate*, *Ficus prostrata*) to male figs is a novel way to for *Apocryptophagus* wasps to adapt to the changes in hosts (i.e., separation of sexual function).

## Material and Methods

### *Ficus auriculata* and associated fig wasps

*Ficus auriculata* Lour. (*Ficus* Sect. Neomorphe) is a common dioecious fig in southern Asia, located in Southwest China. It produces one of the largest figs in this area year around, with diameter averaging around 7 cm. It is pollinated by *Ceratosolen emarginatus* Mayr. It also harbors NPFW in genera *Sycoscapter*, *Philotrypesis*, and *Apocryptophagus*. *Apocryptophagus* sp. wasps oviposit just before the arrival of the pollinating wasps. It induces large gall than pollinator does. Their larvae appeared to feed on proliferating nucellus. *Apocryptophagus* sp. wasps compete with pollinators for floral resources (Weiblen et al. 2001). Yang et al. (2008) found three *Apocryptophagus* species, *Apocryptophagus* sp. 1 can reproduce in both female and male figs of *F. auriculata* in Xishuangbanna forests, but with strong preference to male figs (Yang et al. 2008).

### Specimen collection and morphological study

*Apocryptophagus* wasp specimens were collected from male and female figs of the dioecious *F. auriculata* Lour. during 2007–2009 at five locations in southern China (Danzhou Campus, Hainan University, 19°30′N109°29′E; Yingge Mountain, 19°01′N109°32′E; Changjiang, 19°01′N109°32′E; Jin Tang,18°31′N108°49′E and Xishuang banna arboretum, Yunnan, 21°55′N 101°16′E). The adults from male and females figs were collected and separately stored in 95% ethanol at −20°C. Morphological characters were examined and measured under a Nikon AZ100 microscope system (Tokyo, Japan). Voucher specimens are deposited at Shandong Agricultural University.

### DNA extraction and polymerase-chain reaction amplification

Genomic DNA of each individual was extracted by using DNA Tissue Kit (TransGen Biotech, Beijing, China). Mitochondrial cytochrome c oxidase I (COI) and nuclear ribosomal DNA internal transcribed spacer 2 (ITS2) were successfully identified species of fig wasps (Li et al. 2010; Zhou et al. 2012). A partial of COI and ITS2 sequences were amplified using universal barcoding primers LCO1490 and HCO2198 (Hebert et al. 2003), and ITS2 F:5′-ATTCCCGGACCACGCCTGGCTGA-3′ and ITS2R′:5′-CGCCTGATCTGAGGTCGTC-3′ (White et al. 1990).

Polymerase-chain reaction (PCR) amplification was performed in a volume of 25 μmol/L, containing 2.5 μmol/L 10× buffer, 0.2 mmol/L dNTP, 0.5 μmol/L of each primer, and 0.5 unit of Trans Taq Enzymep (TransGen Biotech, Beijing, China). COI amplification was carried out as the following: 10 min initial denaturation step at 94°C; 94°C for 30 sec, 50°C for 40 sec, 72°C for 60 sec, repeated 35 cycles; then a final elongation step for 10 min at 72°C. ITS2 amplified with 35 cycles of 30 sec at 94°C, 45 sec at 50°C, 75 sec at 72°C.

The PCR products were confirmed using 2% agarose gel, stained with ethidium bromide, and purified using an Easy Pure PCR Purification kit (TransGen Biotech, Beijing, China). Then, the purified products were cloned into pEasy-T1Vector (TransGen Biotech, Beijing, China) and 3–5 positive clones were sequenced by Biosun Sequencing Center, Beijing.

### Sequences and phylogeny analyses

Sequences were eye checked in BioEdit. We also downloaded 78 COI sequences from Genbank and Barcode of Life Data Systems from 31 fig species (Table S1). Fig pollinators (Agaonidae) and NPFW from several subfamilies were considered as outgroup. We also included two genera (*Sycophaga sycomori*, *Idarnes*) of Sycophaginae. All sequences were aligned using ClustalW 1.81. The alignment of COI was confirmed by translating into amino acids in MEGA5. Bayesian inference was employed to estimate phylogenetic relationships (MrBayes 3.12). The best-fitting model of nucleotide substitution was selected in the program of jModeltest based on the Aikake information criterion (Posada 2008). Four Markov Chain Monte Carlo (MCMC) chains were run for 20 million generations and sampled every 1000 generations with the first 20% trees discarded as burn-in. Adequate mixing of the MCMC chain was determined in TRACER version 1.5 (http://tree.bio.ed.ac.uk/software/tracer). Three independent runs were carried out. Heuristic searches under parsimony were conducted with PAUP (Swofford 2002) with 1000 random addition sequence replicates, and bootstrapping with 1000 replicates. Nonparametric bootstrap (BP) value greater than 70% and posterior probability (PP) value greater than 95% were considered as strong support. Divergence time was estimated in BEAST version 1.6.1 (Drummond et al. 2002; Drummond and Rambaut 2007). The GTR+I+G substitution model was employed. The MCMC chain was run for 20 million generations sampled every 1000 generations and the first 20% trees discarded as burn-in. The uncorrelated lognormal model was used to account for rate variation among lineages. *Pegoscapus* fossil (30 million years ago [MYA]) was used to calibrate the date estimation (Rønsted et al. 2005).

## Result

### Morphological examination

We collected 196 specimens from 46 figs, including 31 specimens from three female figs. We examined the morphological diversity under a Nikon SMZ80 microscope and found that seven characters of female *Apocryptophagus* wasps were distinct between wasps from male figs and from female figs. These characters are located in antenna, head, thorax, and wings (details are shown in Table 1 and Fig. S1). For convenience, we named the morphospecies on male fig as *Apocryptophagus* sp. 1, the one on female fig as *Apocryptophagus* sp. 2.

**Table 1:** The description of morphological character of *Apocryptophagus* sp. in *Ficus auriculata* Lour

Character	*Apocryptophagus* sp. 1 (gall fig and seed fig)	*Apocryptophagus* sp. 2 (seed)
Antennal (Fig. S1A and B)	Formula 11263	Formula 1129
	Funicular segments not distinct	Funicular segments subequal in length
	Terminal with one indistinct nipple and without a row of long hair	Terminal with one distinct nipple and a row of long hair
Head and thorax (Fig. S1C and D)	Head surface with dense pits, labiomaxillary complex protrude distinctly	Head surface smooth, labiomaxillary complex not protrude
	Mesosoma with dense puncta in dorsal view pronotum black	Mesosoma smooth in dorsal view pronotum yellow
Wing (Fig. S1E and F)	The length of postmarginal vein is about two times of stigma vein	The length of postmarginal vein is about three times of stigma vein

### DNA sequence analysis

We randomly selected 46 individuals from five geographical locations for DNA barcoding analyses. Of 46 individuals, we successfully amplified COI sequences from 28 individuals and all 46 ITS2 sequences. The lower success rate for amplifying COI fragment was due to the fact that the primers used in this study does not worked well with all samples. The amplified fragment of COI sequences length is 652 bp. We found 33 different haplotype (H1–H33) among 28 individuals (Table 2). The fragment of ITS2 sequence varied in length between two species. The length of *Apocryptophagus* sp. 1 is 373 bp and *Apocryptophagus* sp. 2 is 308 bp, with 12 haplotypes (h1–h12). COI sequences were deposited in GenBank under accession numbers KC421097–KC421131 and for ITS2 KC421132–KC421177.

**Table 2:** Summary of *Apocryptophagus* sp. samples in *Ficus auriculata* and their genetic characteristics

Host fig	Location	Wasp codes	COI haplotype	COI accession number	ITS2 haplotype	ITS2 accession number
Seed	Jintang	ApFJT1	H1/H2	KC421097/KC421098	h1	KC421166
Seed	Jintang	ApFJT2	H3	KC421109	h1	KC421167
Seed	Jintang	ApFJT3	H4	KC421099	h1	KC421168
Seed	Jintang	ApFJT4	H5	KC421104	h1	KC421169
Seed	Jintang	ApFJT5	H6	KC421110	h2	KC421176
Seed	Jintang	ApFJT6	H7	KC421108	h1	KC421170
Seed	Jintang	ApFJT7	H5/H8	KC421105/KC421107	h3	KC421177
Seed	Jintang	ApFJT8	H5	KC421106	h1	KC421171
Seed	Jintang	ApMJT1	H9	KC421100	h1	KC421172
Seed	Jintang	ApMJT2	H10	KC421101	h1	KC421173
Seed	Jintang	ApMJT3	H11	KC421102	h1	KC421174
Seed	Jintang	ApMJT4	H12/H13	KC421111/KC421103	h1	KC421175
Gall	Danzhou	ApFDZ1	H17	KC421116	h5	KC421136
Gall	Danzhou	ApFDZ2	H18	KC421126	h5	KC421137
Gall	Danzhou	ApFDZ3	H19	KC421119	h5	KC421138
Gall	Danzhou	ApFDZ4	H20/H21	KC421130/KC421125	h5	KC421139
Gall	Danzhou	ApFDZ5	H22/H23	KC421120/KC421127	h5	KC421140
Gall	Danzhou	ApFDZ6	H24	KC421117	h11	KC421163
Gall	Danzhou	ApFDZ7	H25	KC421118	h5	KC421141
Gall	Danzhou	ApFDZ8	H26	KC421121	h5	KC421142
Gall	Danzhou	ApMDZ1	H27/H28	KC421122/KC421114	h5	KC421154
Gall	Danzhou	ApMDZ2	**–**	**–**	h6	KC421157
Gall	Xishuang Banna	ApFBN1	H14	KC421112	h4	KC421132
Gall	Xishuang Banna	ApFBN2	**–**	**–**	h8	KC421159
Gall	Xishuang Banna	ApFBN3	H15	KC421113	h12	KC421165
Gall	Xishuang Banna	ApFBN4	H16	KC421115	h9	KC421161
Gall	Xishuang Banna	ApFBN5	**–**	**–**	h5	KC421135
Gall	Xishuang Banna	ApMBN1	**–**	**–**	h5	KC421148
Gall	Xishuang Banna	ApMBN2	**–**	**–**	h5	KC421149
Gall	Xishuang Banna	ApMBN3	**–**	**–**	h5	KC421150
Gall	Xishuang Banna	ApMBN4	**–**	**–**	h5	KC421151
Gall	Xishuang Banna	ApMBN5	**–**	**–**	h5	KC421152
Gall	Yingge Mountain	ApFYGL1	H29	KC421128	h5	KC421143
Gall	Yingge Mountain	ApFYGL2	**–**	**–**	h6	KC421156
Gall	Yingge Mountain	ApFYGL3	**–**	**–**	h5	KC421144
Gall	Yingge Mountain	ApFYGL4	**–**	**–**	h5	KC421145
Gall	Yingge Mountain	ApFYGL5	**–**	**–**	h5	KC421146
Gall	Yingge Mountain	ApFYGL6	**–**	**–**	h5	KC421147
Gall	Yingge Mountain	ApMYGL1	H30	KC421131	h5	KC421155
Gall	Yingge Mountain	ApMYGL2	H31	KC421123	h11	KC421164
Gall	Yingge Mountain	ApMYGL3	**–**	**–**	h4	KC421133
Gall	Yingge Mountain	ApMYGL4	**–**	**–**	h4	KC421134
Gall	Yingge Mountain	ApMYGL5	**–**	**–**	h7	KC421158
Gall	Yingge Mountain	ApMYGL6	**–**	**–**	h8	KC421160
Gall	Changjiang	ApMCJ1	H32/H33	KC421124/KC421129	h11	KC421162
Gall	Changjiang	ApMCJ2	**–**	**–**	h4	KC421153

–, means no acquisition of sequences; Wasp codes: F means female wasp, M means male wasp.

### Phylogenetic analyses and divergence time estimation

**Figure 1:**
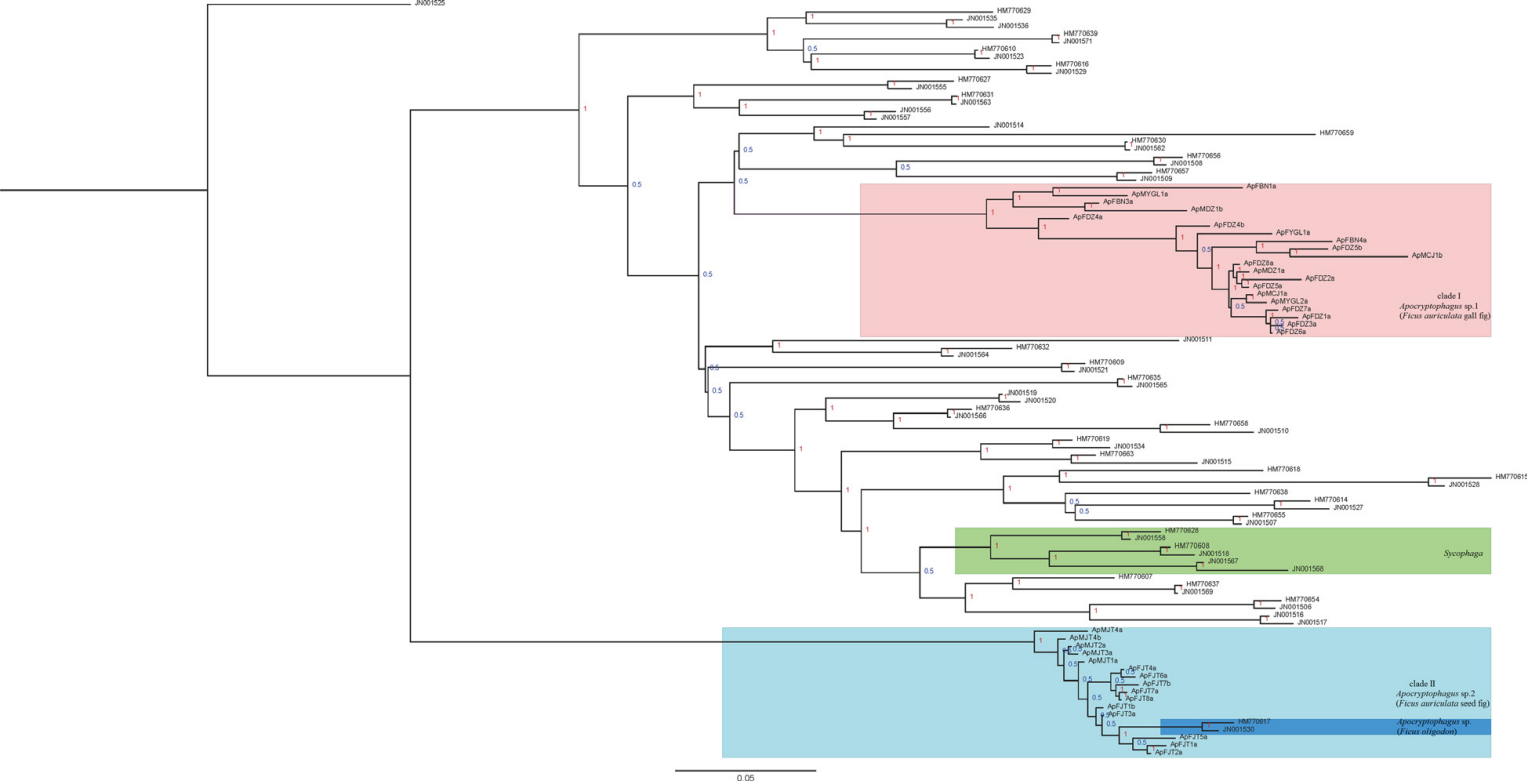
Bayesian tree of relationships among the genus *Apocryptophagus* and the two outgroup taxa based on cytochrome c oxidase I sequences. Values on the nodes are posterior probabilities.

**Figure 2:**
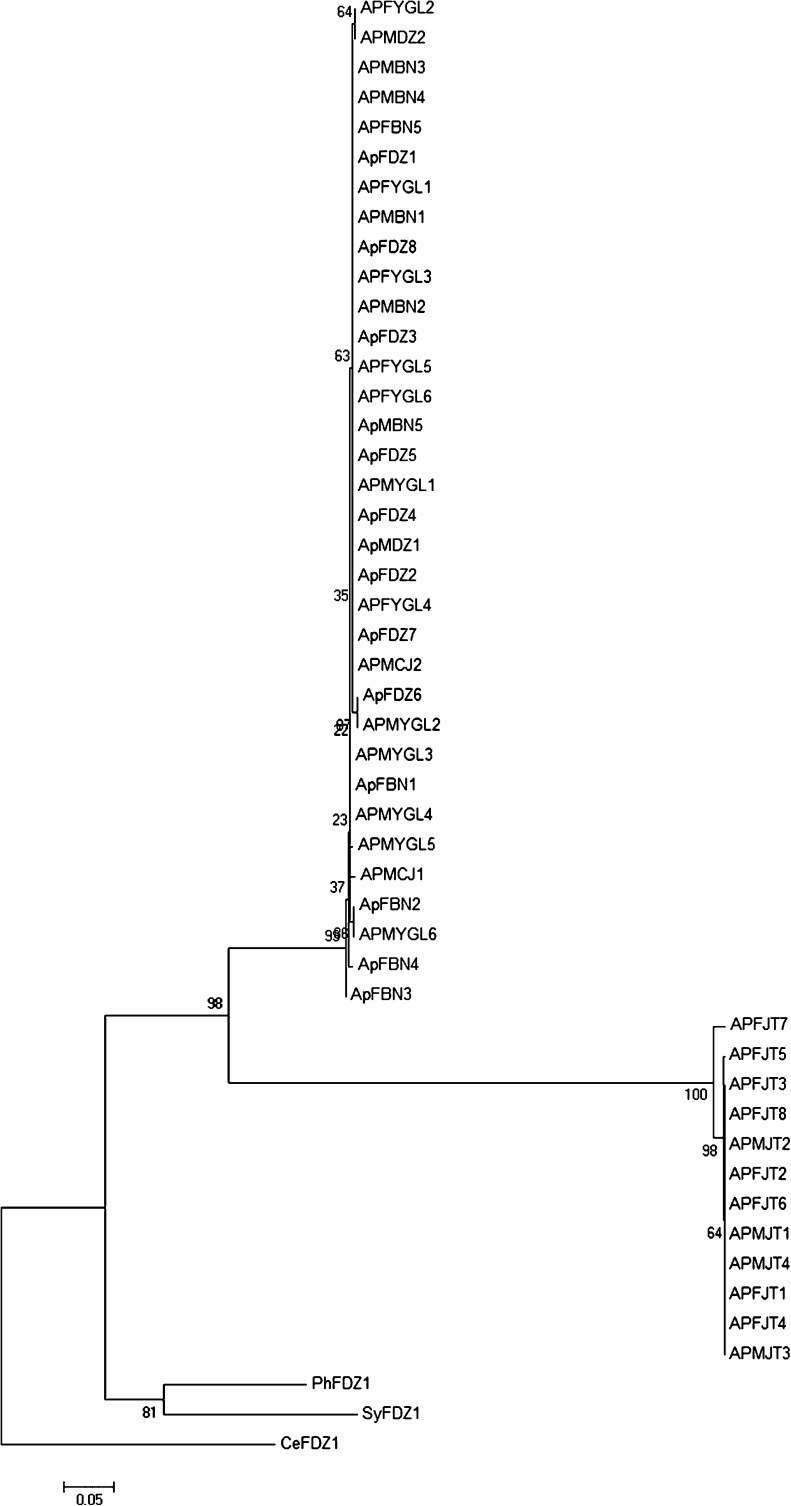
The NJ tree of the genus *Apocryptophagus* based on internal transcribed spacer 2 sequences. Values on the nodes are Bootstrap supports. Pollinating fig wasp *Ceratosolen emarginatus* (CeFDZ1), *Philotrypesis* sp.(PhFDZ1), and *Sycoscapter* sp.(SyFDZ1) as outgroup.

**Figure 3:**
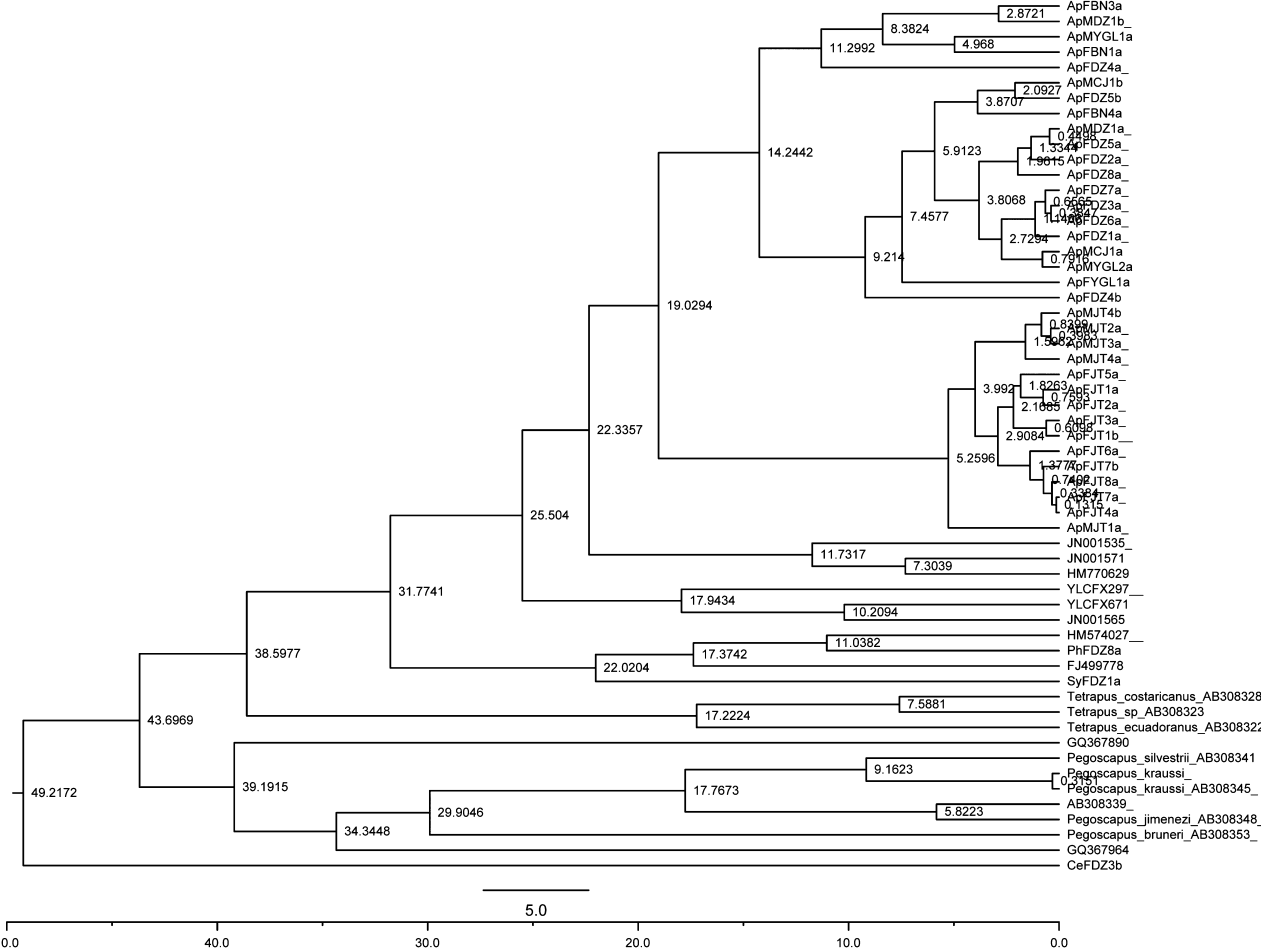
The molecular clock time tree based on COI gene. Based on the *Pegoscapus* fossil (30 MYA) to calibrate the date estimation, *Tetrapus* sp. (AB308323), *Tetrapus ecuadoranus* (AB308322), *Tetrapus costaricanus* (AB308328), *Pegoscapus silvetrii* (AB308341), *Pegoscapus kraussi* (AB308343), *P. kraussi* (AB308345), *Pegoscapus* sp. (AB308339), *Pegoscapus jimenezi* (AB308348), *Pegoscapus bruneri* (AB308353), *Sycophila* sp. 1 (FJ499778), *Pleistodontes xanthocephalus* (GQ367890), *Elisabethiella platyscapa* (GQ367964), *Ormyrus nitidulus* (HM574027), *Apocryptophagus* sp. (YLCFX297-08/YLCFX671-08), and *Sycoscapter* sp. (SyFDZ1)/*Philotypesis* sp. (PhFDZ1)on *Ficus auriculata* were employed for analysis, *Ceratosolen emarginatus* as outgroup.

ACI tests indicate that TIM1+G model (−ln(*L*) = 9533.59, *K* = 203, and AIC = 19473.1798) was selected as the best-fitting model for COI gene. As we expected, the Bayesian tree and maximum parsimony tree based on COI fragments showed similar topologies to previous study (Cruaud et al. 2011) about the phylogenetic relationships of three genera included (*Idarnes*, *Sycophaga*, *Apocryptophagus*). All *Apocryptophagus* wasps were not formed into a monophylogenetic group. The sequences were uploaded to TREEBASE (http://purl.org/phylo/treebase/phylows/study/TB2:S13771). However, *Apocryptophagus* sp. 1 and *Apocryptophagus* sp. 2 were clustered into a well-supported clades (PP = 1; Fig. 1) with clade II having long branch. The mean divergence between two groups is 0.226, which is much large than 0.03, a criteria for delimiting cryptic species in most animal taxa (Haine et al. 2006). Phylogenetic analyses based on ITS2 sequences also showed that *Apocryptophagus* sp. 1 and *Apocryptophagus* sp. 2 formed two distinct clades (BP = 1 and PP = 1) with 1.25 mean genetic distance between two clades (Fig. 2). Thus, DNA barcoding support that the two *Apocryptophagus* morphospecies are two species and that they are not sibling species. Given the *Pegoscapus* fossil record (30 MYA) (Rønsted et al. 2005; Lopez-Vaamonde et al. 2009) and 2.3% mtDNA pairwise divergence/Myr (Brower 1994), we roughly calibrated that two species diverged about 19.2 MYA (Fig. 3).

Within clade II (Fig. 1), the mean divergence is low (0.01). *Apocryptophagus* sp. on figs of *F*. *oligodon* (HM770617/JN001530) from Yunnan province was clustered with all *Apocryptophagus* sp. 2 specimens from Hainan province and shared high similarity (100%). Therefore, we considered them as same species even they lived on different hosts thousands miles away. Clade I consists of all *Apocryptophagus* sp. 1 specimens from all five geographical locations, similar with *Apocryptophagus* sp. on figs of *F. variegate* from Indonesia and *F. prostrata* from China, shared similarity (64%). It seems that *Apocryptophagus* sp. 1 clade further diverged to two groups with mean divergences between two groups being mitochondrial heterogeneity.

## Discussion

*Apocryptophagus*, also known as *Platyneura* in some references, is one-six known genera in subfamily Sycophaginae. It has been shown to be paraphyletic to *Sycophaga*. Most *Apocryptophagus* species are associated with the fig trees of the subgenus *Sycomorus* (Silvieus et al. 2007), with the exception of two species found on *F. orthoneura* (subgenus *Urostigma*, section Urostigma) in southern China. A cophylogenetic analysis of 19 fig species and their associated *Apocryptophagus* wasps was conducted to explore the historical associations. Their study showed that *Apocryptophagus* nonpollinating wasps are not as highly species-specific as *Ceratosolen* pollinators. Five of the 19 fig species (*F. nodosa*, *F. adenosperma*, *F. bernaysii*, *F. congesta*, and *F. hispidioides*) host multiple *Apocryptophagus* wasps. There also have two cases that one *Apocryptophagus* wasp attack more than one fig species. For the cases of more than one *Apocryptophagus* wasps living on same host species, *Apocryptophagus* wasps usually differed in ovipositor length and oviposition timing (i.e., before, during or after pollination) (Kerdelhue and Rasplus 1996). Species with short ovipositors lay eggs prior to pollination when figs are small in diameter, whereas species with long ovipositors lay eggs after pollination when figs are larger (Weiblen and Bush 2002). There is tight correlation with the ovipositor length with the fig size when *Apocryptophagus* oviposit. Evidence that multiple parasite lineages colonized the same fig species independently (Weiblen and Bush 2002).

In host–parasites interaction, hosts usually dominate this interaction and there is strong natural selection for parasites to adapt to the changes in hosts. If there are major phenotype changes in hosts make it unsuitable place for parasites, parasites either go extinction or switch to other hosts that have similar habitats and less competition (Silvieus et al. 2007; Mcleish et al. 2010). To escape the disadvantage or sanction impact of the host, the exploiter *Apocryptophagus* wasps can preferably adapt to exploiting each sex of the figs, by changing their oviposition, niche shift, and habitat (Wang et al. 2010). For *Apocryptophagus* wasps, under frame of morphology difference between male and female figs, we found the galls only closed to the ostiole of female figs; however, the galls distributed covering male figs. There was no dissimilarity in the thickness or other structures between male and female figs. Our examination suggested that spatial niche partitioning may sufficiently favor exploiters in exploiting the female resource, and there was no competition with pollinators or other parasites. Unfortunately, in our fieldwork, we did not collect the *Apocryptophagus* wasps on male figs in Jintang location, the galls had been an empty house without wasps information. The thickness of the fig wall and the timing of oviposition with respect to fig development appear to be traits that could facilitate a host shift. Sister group comparisons showed that there is a tendency for *Apocryptophagus* to shift to figs with similar wall thickness (Weiblen and Bush 2002).

Reciprocal evolution between fig and fid wasp is a typical case of diversifying coevolution, in which the interaction cause at least one of the species to become subdivided into two or more reproductively isolated populations(Thompson 1989). On the basis of the morphology and DNA barcoding from partial sequences of COI and ITS2, we found that there are two nonsibling *Apocryptophagus* species living on male and female *F. auriculata* figs, respectively. *Apocryptophagus* sp. 2 attack both *F. auriculata* (female figs) and *F*. *oligodon* figs (male figs). However, we have not found that *Apocryptophagus* sp. 1 can live or be reared from fig species other than *F. auriculata* fig Peng et al. (2005) studied on the population dynamics of *Apocryptophagus* sp. on dioecious *F. auriculata* fig, and found the reproduction of *Apocryptophagus* sp. on female syconia was limited. Their results suggested that *Apocryptophagus* sp. preferred ovipositing male syconia to female syconia. Only when there were few or no male syconia available did it shift its reproduction to female syconia (Peng et al. 2005). In addition, *Apocryptophagus* sp. 1 only exists in the male fig in other locations of Hainan and Xishuang banna arboretum, Yunnan, there is no reproduction shift to female syconia, even less male figs on a tree. Our *Apocryptophagus* sp. 1 wasps are different from Peng's fig wasp species in the length of ovipositor, more similar to *Apocryptophagus* sp. on the figs of *F. variegate* and *F. nodosa* from Indonesia. Above all, this suggests that occurrence of *Apocryptophagus* sp. 1 in *F. auriculata* male syconia is likely to be a host shift event (Cook and Segar 2010), in ecologically associations similar to the yucca–yucca moth mutualisms (Kawakita and Kato 2006).

Stability of this mutualism depends on the relative allocation of floral resources to pollen, seeds, and pollinators. Given that pollinators also eat seeds, there is potential evolutionary conflict between seed production and seed consumption (Cook and Rasplus 2003). In functionally dioecious figs, this conflict is resolved by segregating the production of seeds and pollinators in two types of figs on separate plants (Weiblen et al. 2001). Molecular phylogeny suggested that dioecy arise independently multiple times in several lineages (Weiblen 2001; Jousselin et al. 2003). A key of maintaining mutualism in dioecious figs is that female figs can regularly deceive pollinators into visiting despite the absence of any reproductive reward (Grafen and Godfray 1991). Chemical volatiles are the primary cues that attract highly species-specific pollinators species to receptive figs (Hossaert-McKey et al. 1994; Grison-Pige et al. 2002). The same chemical volatiles are used for NPFW such as *Apocryptophagus* to search for host figs. Thus, male and female figs should also be visited comparable amount of times by *Apocryptophagus*. This is true for NPFW on few fig species that have been investigated including *Apocryptophagus* sp. 1 (Proffit et al. 2007). However, we do not know whether *Apocryptophagus* sp. 2 has developed ability to distinguish female figs from male figs and only visit female *F. auriculata* fig. If it were, pollinator might also have a chance to develop or have developed his ability to distinguish the sexual figs. In that case, the fig–fig wasp mutualism on *F. auriculata* is on the eve of collapse.

## Data Accessibility

Taxa information: Table 1. DNA sequences: Genbank accessions KC421097–KC421131 and KC421132–KC421177. Phylogenetic data: TreeBASE Study accession no. S13771.

## Supporting Information

**Table S1.** Fig wasps and outgroup species downloaded in this study.

**Figure S1.** Morphological images of female wasps of *Apocryptophagus* sp. 1 and *Apocryptophagus* sp. 2 in *Ficus auriculata*. Antennal of female: (A) *Apocryptophagus* sp. 1, (B) *Apocryptophagus* sp. 2. Dorsal habitus of female: (C) *Apocryptophagus* sp. 1, (D) *Apocryptophagus* sp. 2. Forewing of female: (E) *Apocryptophagus* sp. 1, (F) *Apocryptophagus* sp. 2.
