# Supplementary figures and images for: Host sex-specific parasites in a functionally dioecious fig: a preference way of adaptation to their hosts
Source: Ecol Evol. 2013 Jul 30;3(9):2976–84. doi: 10.1002/ece3.682 (PMC3790544; doi:10.1002/ece3.682)

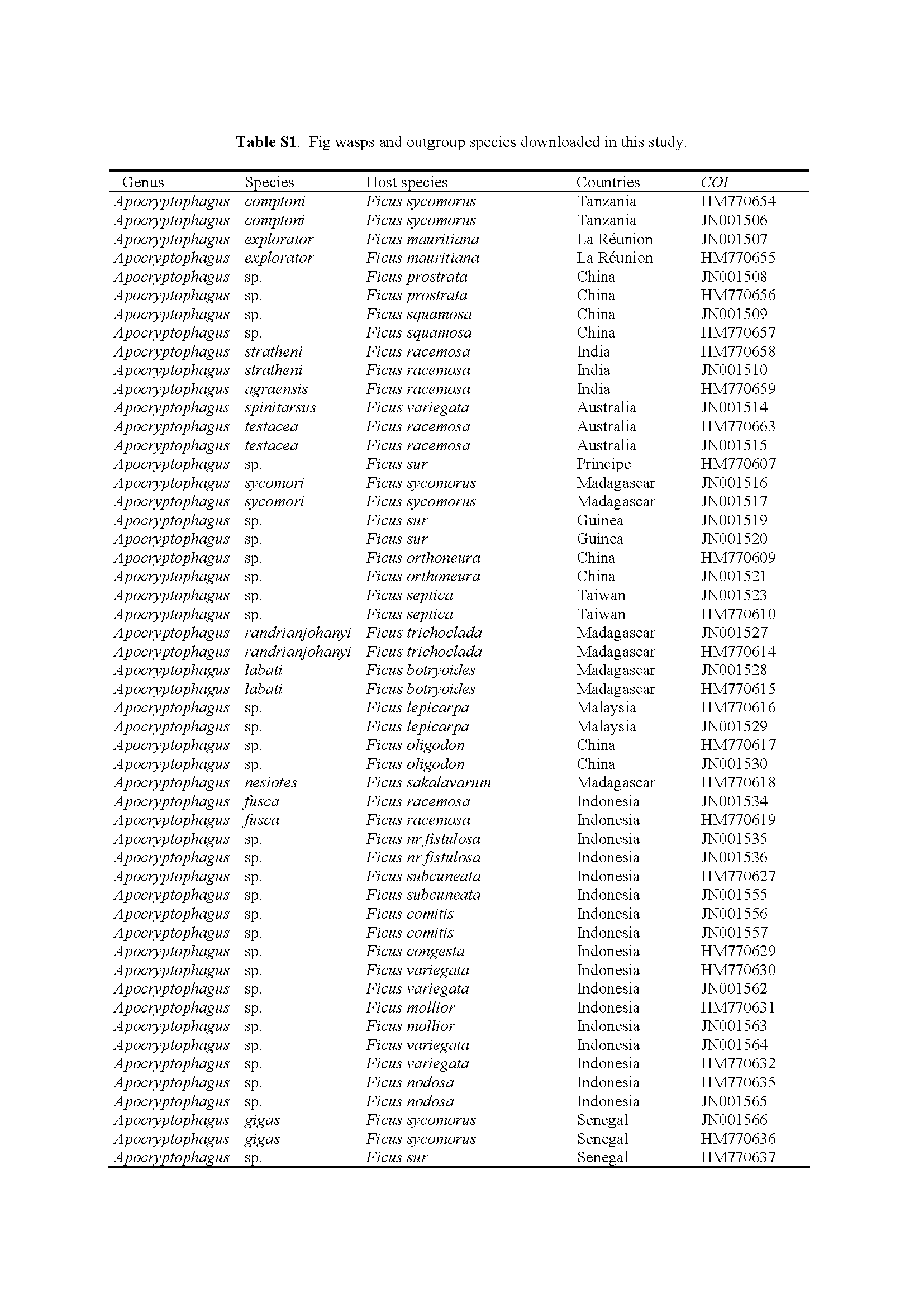

Supplement: Supplementary file 1 [file ece30003-2976-SD1.tiff]

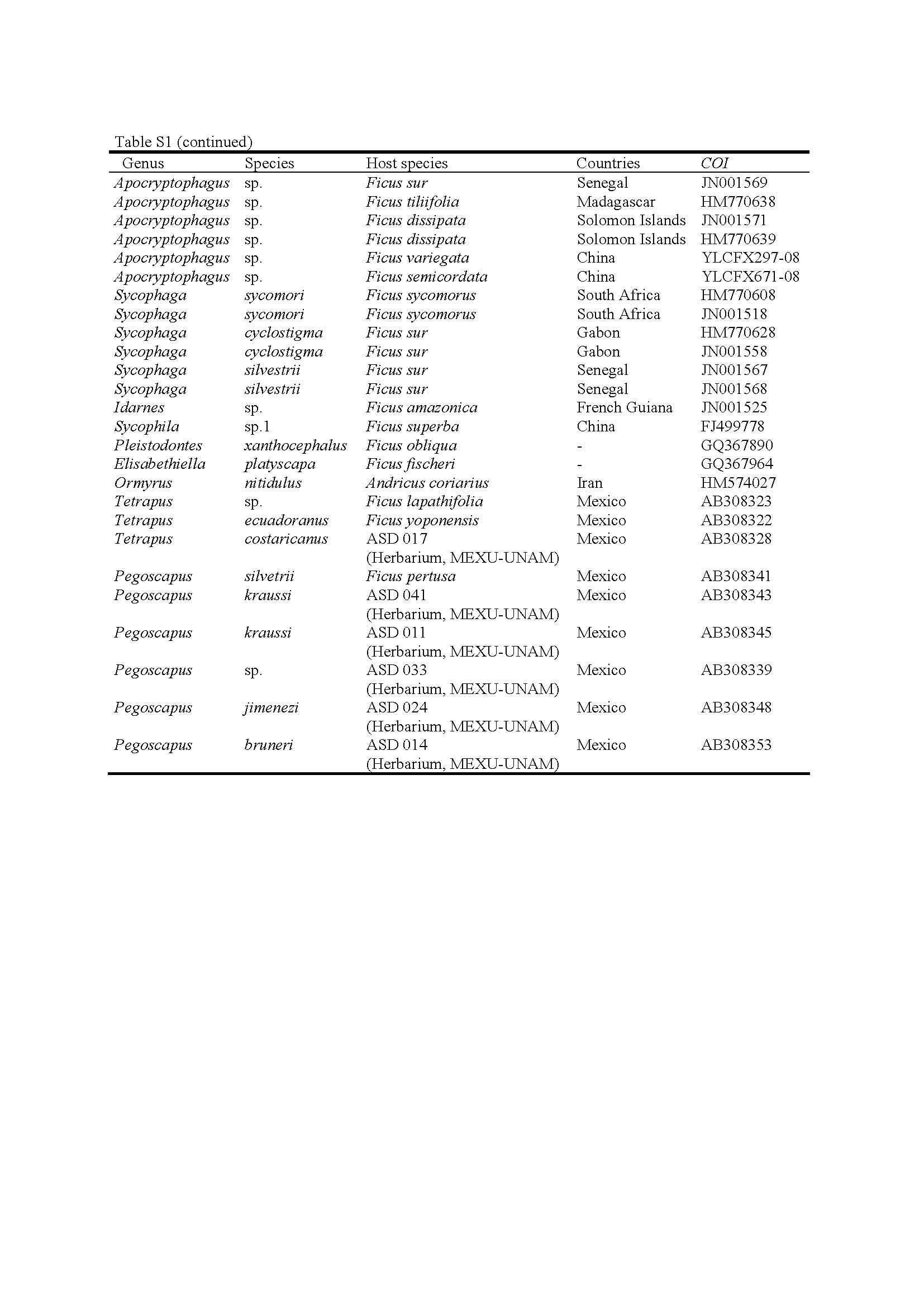

Supplement: Supplementary file 2 [file ece30003-2976-SD2.tiff]

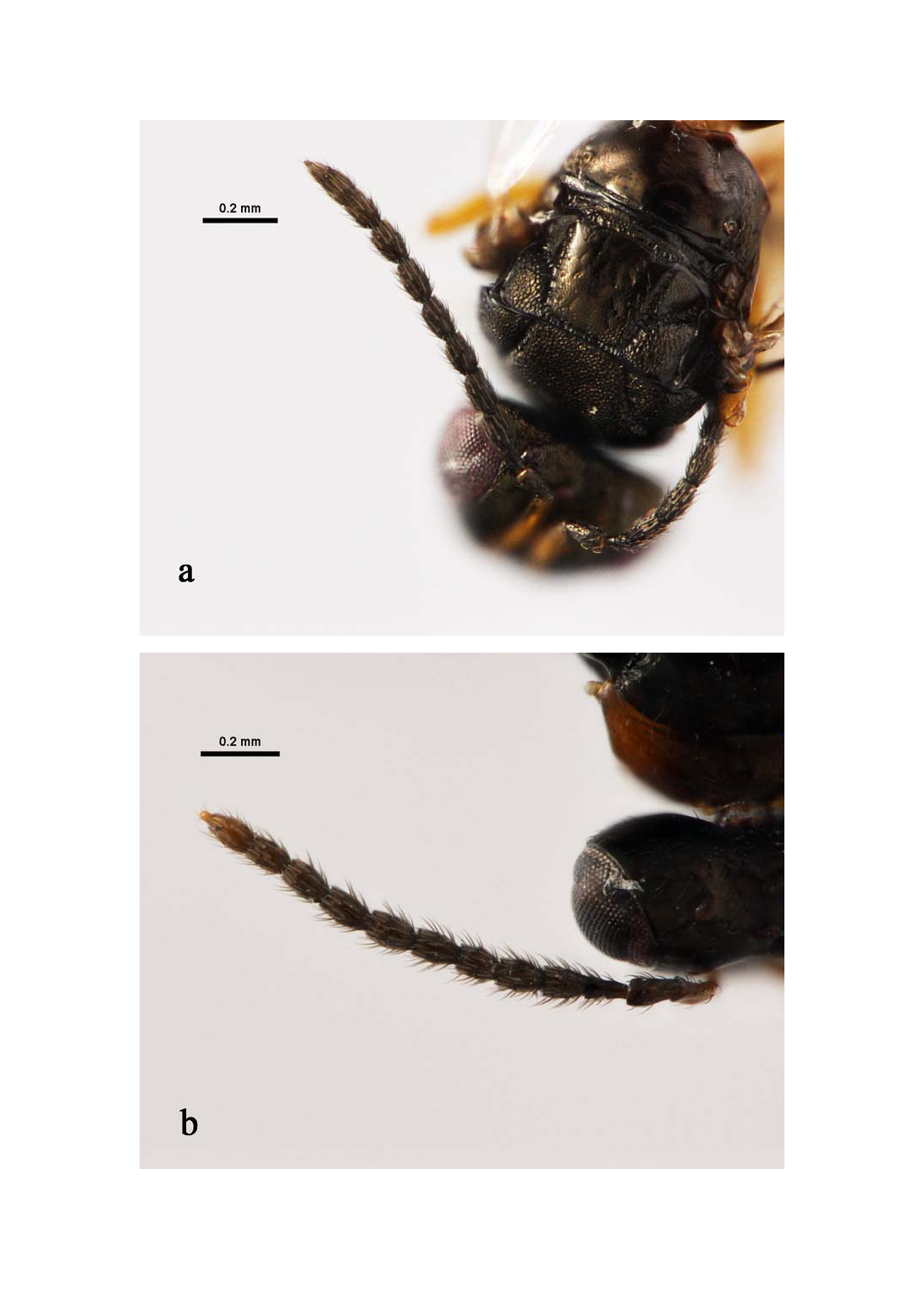

Supplement: Supplementary file 3 [file ece30003-2976-SD3.tif]

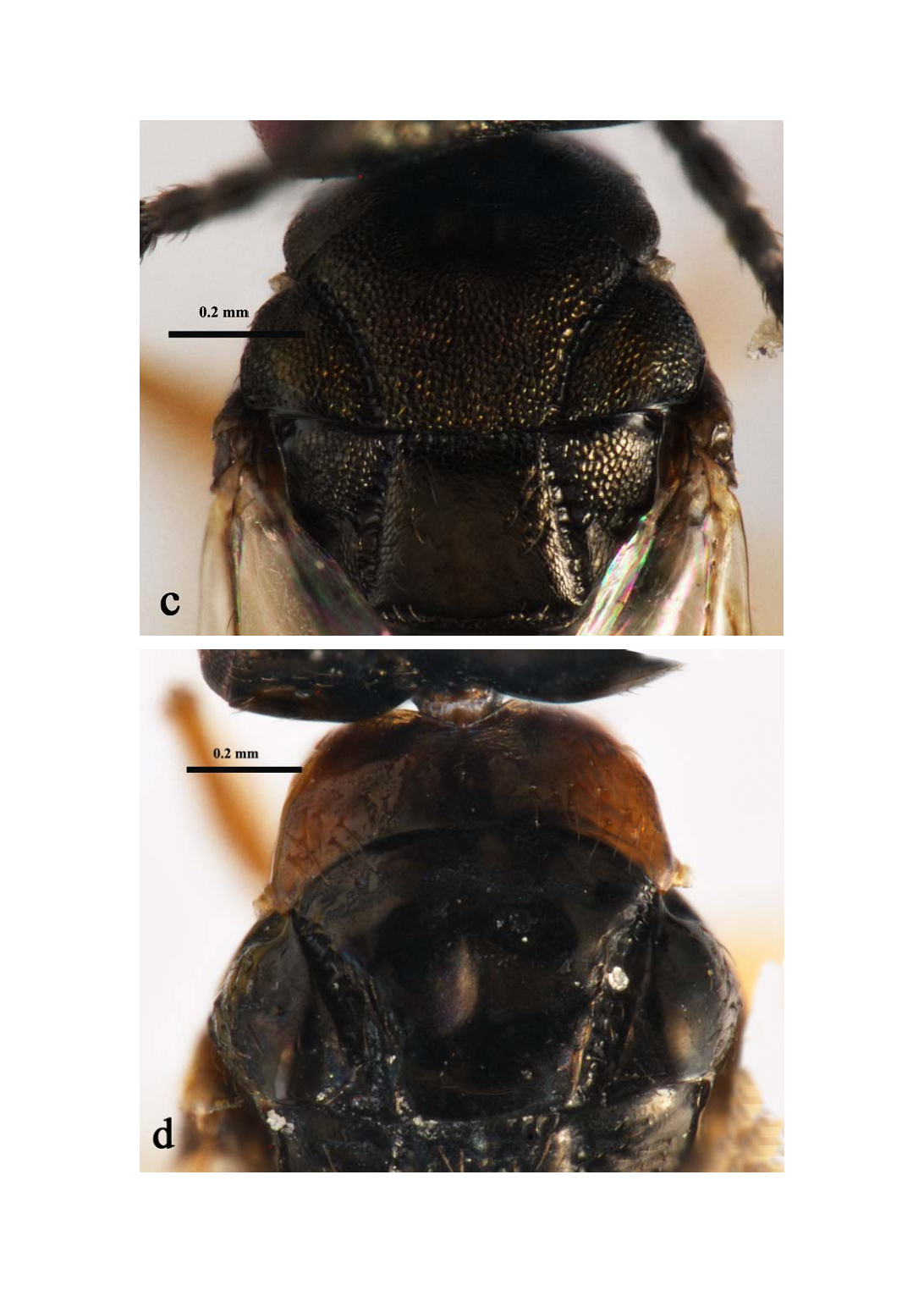

Supplement: Supplementary file 4 [file ece30003-2976-SD4.tif]

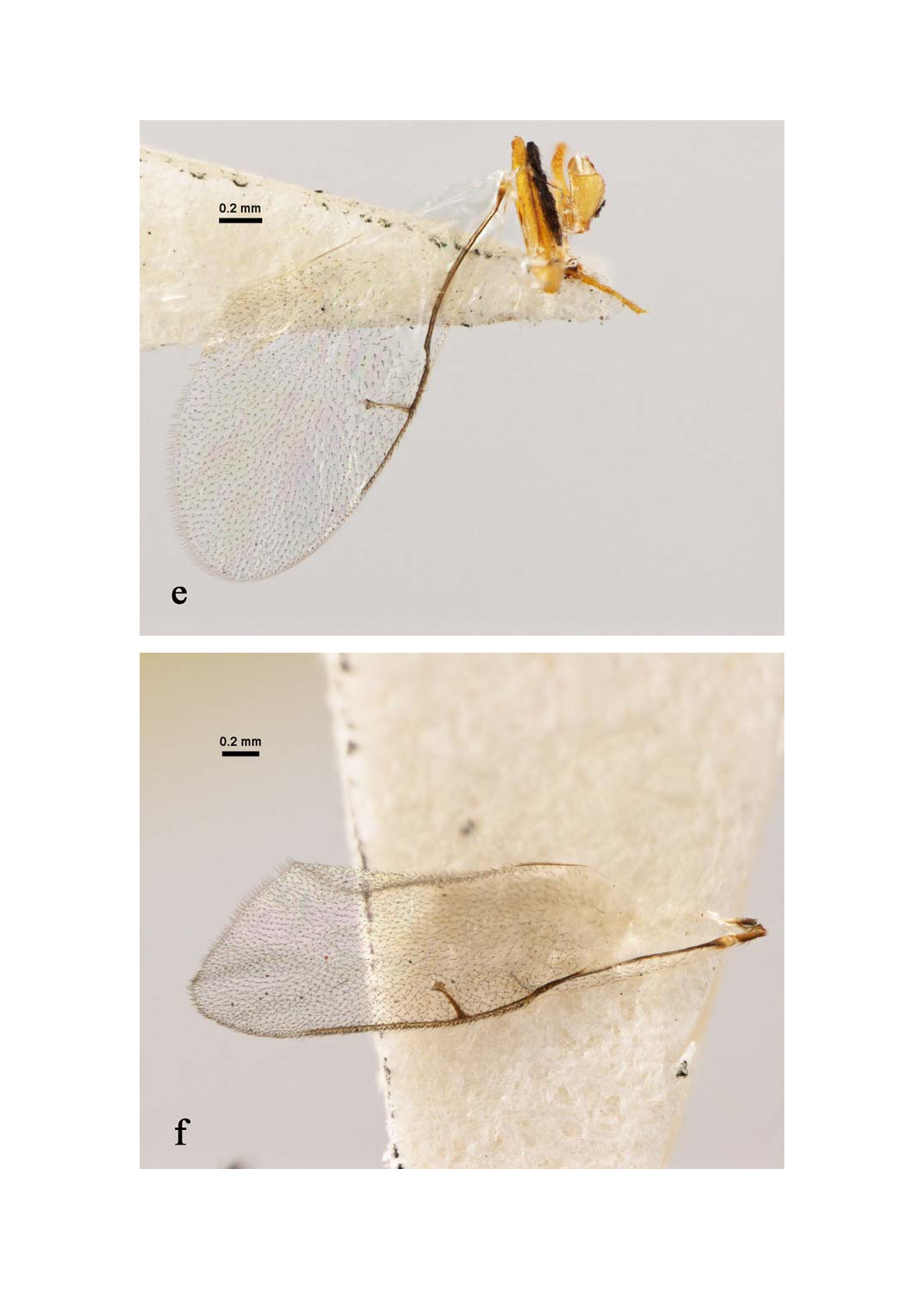

Supplement: Supplementary file 5 [file ece30003-2976-SD5.tif]
